# The Effect of Injection Parameters on Drug Distribution for Spinal Anesthesia: A Numerical Approach

**DOI:** 10.3390/jcm14176236

**Published:** 2025-09-03

**Authors:** Mürsel Kahveci, Levent Uğur

**Affiliations:** 1 Anesthesiology and Reanimation, Amasya Training and Research Hospital, Amasya University, Amasya 05100, Turkey; drmurselkahveci@yahoo.com; 2 Department of Mechanical Engineering, Faculty of Engineering, Amasya University, Amasya 05100, Turkey

**Keywords:** cerebrospinal fluid, computational fluid dynamics, biomechanics, intrathecal drug delivery

## Abstract

**Background:** Spinal anesthesia is a widely used technique for pain control in surgical procedures, requiring effective drug distribution within the cerebrospinal fluid (CSF) for optimal outcomes. The distribution is influenced by injection parameters such as needle diameter and injection speed, which, if not optimized, can reduce efficacy or cause side effects. This study investigates how these parameters affect drug distribution in the CSF using computational fluid dynamics (CFD). **Material Methods:** An anatomically accurate three-dimensional model of the CSF space was created using MRI data. Simulations were performed using three needle tips (22 G, 25 G, 27 G) and different injection rates at the L4–L5 vertebral level. The model included physiological CSF oscillations from cardiac and respiratory cycles. Drug dispersion was analyzed in terms of spatial distribution and concentration changes over time. **Results:** The findings obtained show that the combination of a large-gauge needle (22G) and high injection speed provides wider distribution within the CSF and more effective transport to the cranial regions. On the other hand, with a small-gauge needle (27G) and low injection speed, the drug remained more localized, and access to the upper spinal regions was limited. Additional parameters such as injection duration, direction, and flush applications were also observed to significantly affect distribution. **Conclusions:** CFD modeling reveals that injection parameters significantly affect drug dispersion patterns in spinal anesthesia. Optimizing these parameters may improve therapeutic outcomes and reduce complications. The model provides a foundation for developing personalized intrathecal injection protocols.

## 1. Introduction

Cerebrospinal fluid (CSF) is found in two primary areas: the intracranial space and the spinal canal. CSF is a dynamic and complex fluid that facilitates the transport of dissolved substances around the brain and within the ventricles. In healthy adults, the total volume of CSF ranges from 250 to 400 mL, and it has viscosity and density values similar to those of water [1,2]. It exhibits an oscillatory motion at speeds ranging from 0 to 15 cm/s due to the effects of cardiac and respiratory cycles [3]. This dynamic structure of the CSF plays a critical role in drug transport and distribution. As highlighted by recent studies, CSF motion is governed not only by bulk flow but also by filtration and perivascular mechanisms, which together facilitate the transport of substances throughout the CSF system [4].

These dynamic properties play an important role in the effectiveness of intrathecal drug administration. In this context, understanding the properties and transport capacity of CSF is critical to ensuring the proper distribution of intrathecal drugs. Spinal anesthesia is a method of administering local anesthetics into the CSF to provide effective pain control during surgical procedures [5]. This technique typically creates sensory and motor blockage in body segments below the umbilicus and can be optimized by considering the effects of cardiac and respiratory cycles on the CSF. Although the complex anatomy and physiology of the CSF make it difficult to accurately predict drug distribution patterns, intrathecal drug administration offers an important treatment approach in areas such as chronic pain management, spasticity treatment, and chemotherapy for certain types of cancer [6]. In this context, understanding drug dynamics within the CSF is of great importance in terms of increasing therapeutic efficacy and minimizing side effects [4].

Drug distribution in CSF depends on many parameters, such as needle diameter, injection speed, and level. For example, wider diameter needles (e.g., 22G) allow for rapid drug distribution, while smaller diameter needles (e.g., 27G) provide controlled distribution [7]. Kuttler et al. developed and applied simplified spinal CSF geometry to model CSF transport. The analysis provided some basic information about drug distribution in the CSF after lumbar puncture injection [8]. Another study examined intrathecal drug delivery with idealized 3D elliptical geometry to evaluate the potential effects of flow rate and catheter size and orientation [9]. In addition, it is known that the injection speed also affects the homogeneous distribution of the drug and that high speeds in particular can cause turbulence [10]. For these reasons, it is important to optimize these parameters in order to increase the effectiveness of spinal anesthesia. These studies have highlighted the need for systematic investigation of these factors by demonstrating that injection parameters significantly affect the spatial and temporal distribution of drugs.

Computational fluid dynamics (CFD) has emerged as a powerful tool for simulating fluid flow and drug distribution in biological systems, including the BOS [11]. Numerical modeling studies are an effective tool for investigating the effects of these parameters on drug diffusion within the CSF. CFD methods, in particular, provide the ability to analyze the velocity and concentration distributions that occur during injection in detail. This allows for a better understanding of irregularities in drug diffusion and turbulence effects.

In the literature, advanced magnetic resonance imaging (MRI) and CT-based three-dimensional models are used to increase anatomical accuracy in simulations of intrathecal drug distribution. However, modeling the multiple variables that affect CSF dynamics remains a significant challenge. In particular, studies are ongoing to more accurately model the effect of respiratory and cardiac cycles on the turbulent flow within the CSF and the contribution of turbulence to drug distribution. Today, advanced particle tracking methods and numerical analysis techniques offer the opportunity to examine in greater detail the effects of injection rates and needle diameters on distribution. The novelty of this study lies in the combination of anatomically accurate three-dimensional models with time-dependent analyses to optimize drug distribution within the CSF, and the systematic evaluation of the effects of injection parameters on CSF flow. This approach aims to enhance the efficacy of spinal anesthesia and intrathecal drug administration, thereby achieving more reliable outcomes in clinical procedures.

In the literature, there appears to be no comprehensive study that systematically evaluates the effects of injection parameters (needle diameter, injection speed, etc.) on CSF flow with a focus on optimizing spinal anesthesia procedures. To address this gap, this study utilized MRI images to create a three-dimensional CSF model with high anatomical accuracy and investigated the effects of different needle gauges (22G, 25G, 27G) and injection speeds (0.01 m/s, 0.05 m/s, 0.1 m/s) were investigated using numerical modeling. Additionally, time-dependent dynamic analyses were performed to understand the effects of cardiac and respiratory cycles on CSF flow and drug distribution. The findings aim to contribute to making spinal anesthesia procedures safer and more effective.

## 2. Materials and Methods

This section provides the physical models, mathematical models, and numerical methods used in the analysis. The simulations were designed to evaluate velocity contours at the injection site, time-dependent intrathecal drug distribution patterns, and average drug concentration percentages at various spinal levels.

### 2.1. Geometric Model

**Figure 1:**
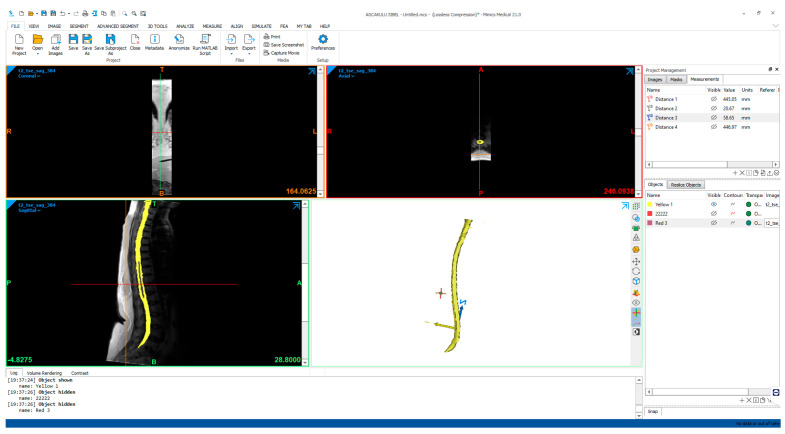
Creation of a CSF model from an MRI image.

In our study, Magnetic Resonance Imaging (MRI) data obtained from a healthy 30-year-old individual was used to create an anatomically realistic CSF system model. MRI slices were obtained using a GE Revolution CT 128-Slice (GE Healthcare, Chicago, IL, USA) device with a slice thickness of 0.625 mm in the axial plane. The acquired image data were processed using Materialise MIMICS 17 (Materialise NV, Leuven, Belgium), an advanced medical image processing software, to provide a three-dimensional reconstruction of the CSF system, as shown in Figure 1. During this process, segmentation was performed to create a three-dimensional model with high anatomical accuracy from the MRI data. During the segmentation process, a threshold range of 69–693 Hounsfield units (HU) was used to define the boundaries of the CSF system and separate it from surrounding tissues.

**Figure 2:**
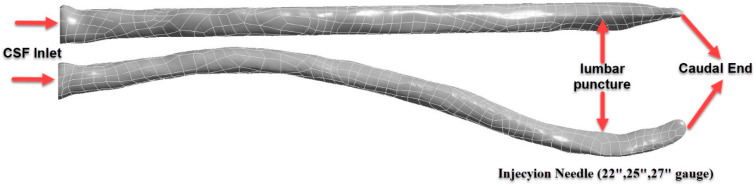
Optimized 3D model of the CSF structure obtained via reverse engineering in Geomagic Studio.

The obtained three-dimensional model was optimized using the reverse engineering software package Geomagic Studio 12.0 (Geomagic, Cary, NC, USA) in order to achieve a more detailed CSF structure. During this process, the surface details of the CSF system were improved and the anatomical accuracy of the model was increased. As shown in Figure 2, the effects of intrathecal injections performed at the L4–L5 vertebral level on drug distribution within the CSF were investigated by transferring the data to the ANSYS Workbench (ANSYS Inc., Canonsburg, PA, USA) environment.

To evaluate the impact of injection parameters on intrathecal drug distribution, a total of nine simulation scenarios were constructed, as outlined in Table 1. Three different needle gauges (22G, 25G and 27G) were each tested under three distinct injection velocity conditions. In all simulations, the total volume of injected drug was kept constant to isolate the effects of needle diameter and injection speed. Importantly, the injection duration was standardized across all experiments, and injection velocity was adjusted accordingly based on the inner diameter of each needle type. This approach enabled a systematic comparison of how varying fluid dynamics influence spatial and temporal patterns of drug dispersion within the CSF.

### 2.2. Mathematical Model

The flow and drug transport within the CSF were modeled using fluid mechanics equations and transport-diffusion equations. Assuming that the CSF is an incompressible and Newtonian fluid, the Navier–Stokes and continuity equations were used:

(1) ∇.u=0

(2) $$\rho \left(\frac{\partial v}{\partial t}+v.\nabla v\right)=-\nabla P+\mu \nabla^{2}v+F$$

Here, ρ represents the density of the fluid, v represents the velocity vector, P represents the pressure, μ represents the dynamic viscosity, and F represents the external forces.

The transport of drugs within the CSF was modeled using the advection-diffusion equation:

(3) $$\frac{\partial C}{\partial t}+v.\nabla C=D\nabla^{2}C$$

Here, C is the drug concentration and D is the diffusion coefficient. To capture the high velocity gradients at the injection site, the equations were solved using a second-order UPWIND method.

**Figure 3:**
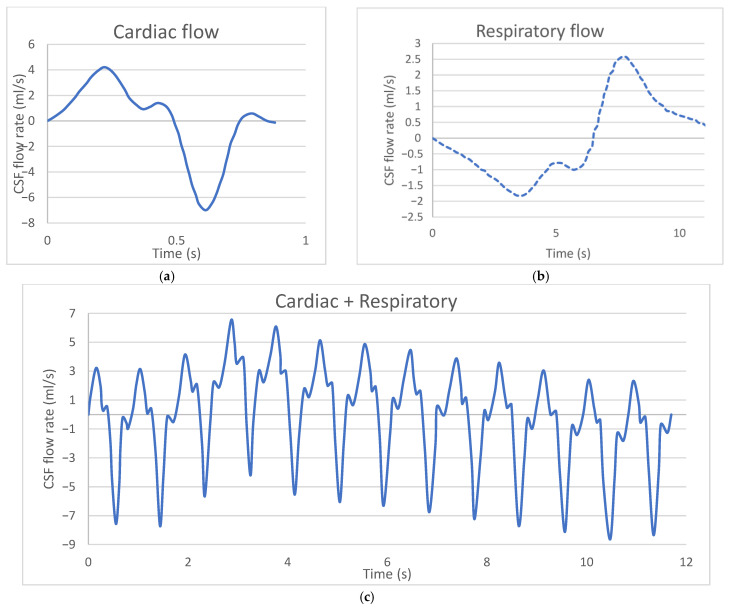
(**a**) Cardiac flow waveform, (**b**) Respiratory flow waveform, (**c**) Final flow waveform obtained by combining the respiratory and cardiac flow waves [13,14].

In this study, analyses were performed in the supine position to represent a typical patient position for Lumbar Puncture (LP) injection. A oscillating velocity boundary condition was applied at the caudal opening to simulate CSF oscillations around the spinal cord. The CSF flow velocity waveform was modeled with a pulse volume of 1.0 mL at the C2–C3 vertebral level, and the ventricular CSF production rate was determined to be 0.4 mL/min (~576 mL/day) according to the literature [12]. Non-sliding boundary conditions were assigned to the model walls (dural, pial, and intraventricular spaces). CSF was modeled as an incompressible fluid identical to water at room temperature, with a density of 998.3 kg/m^3^ and a viscosity of 0.89 mPa·s. The CSF flow waveform measured at the C2–3 vertebral level using Phase-Contrast Magnetic Resonance Imaging (PCMRI) was used for the time-dependent inlet velocity profile of CSF flow. Figure 3a shows the cardiac flow waveform, Figure 3b shows the respiratory flow waveform, and Figure 3c shows the final flow waveform obtained by combining the respiratory and cardiac flow waves [13,14]. This modeling approach ensured that drug transport within the CSF was simulated as closely as possible to reality, and the effects of injection parameters on drug distribution were examined in detail.

### 2.3. Numerical Model

Computational analyses were conducted using ANSYS Fluent 2022R1 (Canonsburg, PA, USA). A laminar viscous flow model was applied to solve the oscillatory flow field within the CSF. Mass and momentum conservation equations were solved with a stringent convergence criterion. Specifically, the solution was considered converged when the normalized absolute difference between two successive iterations of any variable satisfied the inequality given in Equation (4):

(4) $$\left|\frac{\delta^{n+1}\left(i,j,k\right)-\delta^{n}\left(i,j,k\right)}{\delta^{n+1}\left(i,j,k\right)}\right|<10^{-6}$$

In this equation, *n* and *n* + 1 denote two successive iterations, *δ* represents any flow variable, *i*, *j* and *k* correspond to the grid positions in the *x*, *y* and *z* directions, respectively. The use of this strict convergence criterion ensured the accuracy and reliability of the simulation results.

All simulations were executed in parallel mode on a computing system equipped with 32 GB of RAM and 12 processors. Approximately three days of computation time were required to simulate a 6-min physical flow process.

For the pressure discretization, the PRESTO! (Pressure Staggering Option) scheme was employed, while the flow equations were solved using the PISO (Pressure-Implicit with Splitting of Operators) algorithm. Second-order upwind and first-order upwind schemes were applied for the momentum and volume fraction equations, respectively. Relaxation factors were set to their default values. An implicit formulation was used for volume fraction calculations, and the dispersed model was selected for phase interface modeling.

**Figure 4:**
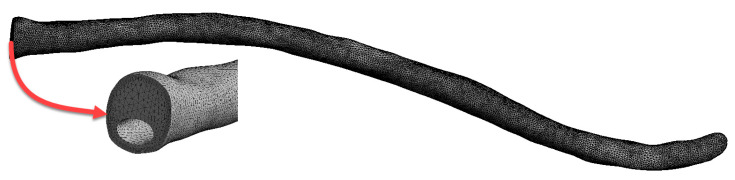
Mesh structure.

To minimize interfacial errors caused by numerical diffusion, mesh refinement was performed near the injection region. A fixed time step of 0.01 s and a maximum element size of 0.5 mm were defined for each simulation. To accurately represent the complex geometry of the CSF model, a tetrahedral mesh structure was generated (Figure 4). The use of tetrahedral elements enabled higher-resolution analysis in the irregular and intricate CSF domain, thereby enhancing overall solution accuracy.

### 2.4. Verifcation of Numerical Results

**Figure 5:**
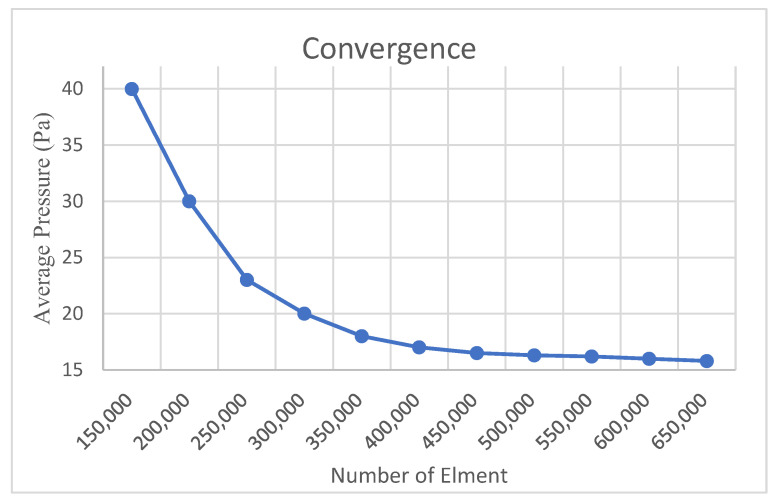
Convergence study results in CFD.

To evaluate the accuracy of the numerical results obtained in this study, a mesh independence analysis was conducted. Comparative simulations were performed using models with varying numbers of mesh elements to assess the influence of mesh density on the solution. The convergence behavior of the solution was evaluated by tracking the variation in average pressure (Pa), and the resulting convergence curve is presented in Figure 5.

As illustrated in Figure 5, simulations performed with mesh densities ranging from 150,000 to 650,000 elements revealed a rapid decrease in the average pressure value, with the rate of change falling below 1% beyond approximately 500,000 elements. This trend indicates that the numerical solution becomes less sensitive to further mesh refinement and demonstrates a convergent behavior.

The average pressure value used for this evaluation was calculated at the final time step of the simulation and represents the average pressure on all internal wall boundaries of the system. This definition ensures a global assessment of pressure stability and numerical consistency.

Based on this analysis, it was determined that a mesh containing approximately 500,000 to 550,000 elements provides sufficient accuracy while maintaining computational efficiency. These findings support the reliability of not only the average pressure predictions but also other flow-related parameters computed within the domain, thereby confirming the numerical stability of the model.

Consequently, the mesh configuration used in this study was deemed adequate to accurately simulate the physical behavior of intrathecal drug distribution within the CSF system.

## 3. Results

The analysis results indicate that injection parameters play a critical role in determining both the local concentration and the direction of drug transport.

### 3.1. Velocity Distribution and Inlet Profile Analysis

**Figure 6:**
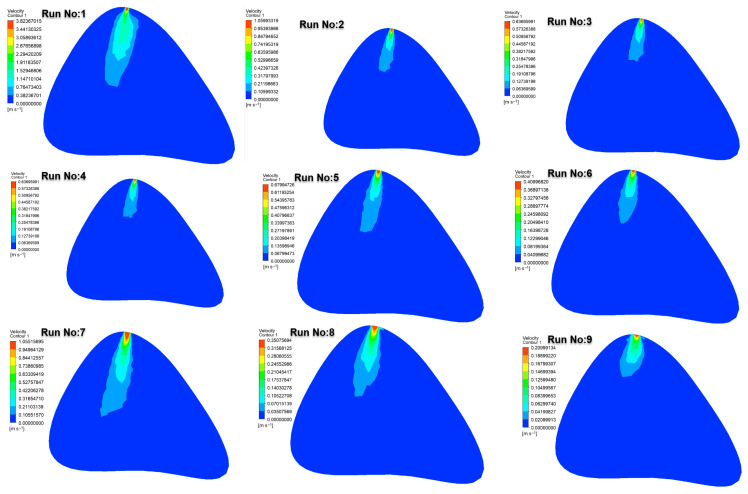
Velocity contours at the 10th second in the lumbar puncture section for all test cases.

Figure 6 illustrates the velocity distributions obtained at the 10th second of injection in the lumbar puncture region. At higher injection velocities (e.g., Run No: 1–3.6 m/s), abrupt and sharp velocity gradients were observed near the injection site, generating a driving force that initiated drug transport. The highest velocity zones were detected in cases involving injections through a 22G needle.

In contrast, at lower injection velocities (e.g., Run No: 9–0.21 m/s), the drug entered the cerebrospinal fluid (CSF) with lower momentum, resulting in more restricted distribution within the CSF. These findings demonstrate the strong influence of injection velocity on both the initial momentum and the extent of drug dispersion. It should also be noted that in intermediate cases (e.g., Runs 4–6 with the 25G needle), the velocity zones exhibited partially overlapping features with those observed in the 27G group. This similarity is attributable to the lower kinetic energy imparted to the fluid at reduced injection speeds, which diminishes the gradient differences expected between medium- and fine-gauge needles. Thus, the velocity distribution is not solely determined by the needle gauge but by the combined effect of gauge and injection velocity.

### 3.2. Time-Dependent Distribution of Drug Concentration: C7–T1 and T12–L1 Levels

To evaluate the effectiveness and directionality of intrathecal drug dispersion, concentration levels were specifically examined at two anatomically and physiologically relevant spinal regions: C7–T1 and T12–L1. The C7–T1 region represents the upper cervical spinal cord and serves as an indicator of cranial drug transport, which is critical for therapeutic targeting of central nervous system structures. Conversely, the T12–L1 level, located near the injection site, reflects localized drug accumulation and is important for assessing the immediate efficacy and retention of the administered agent.

**Figure 7:**
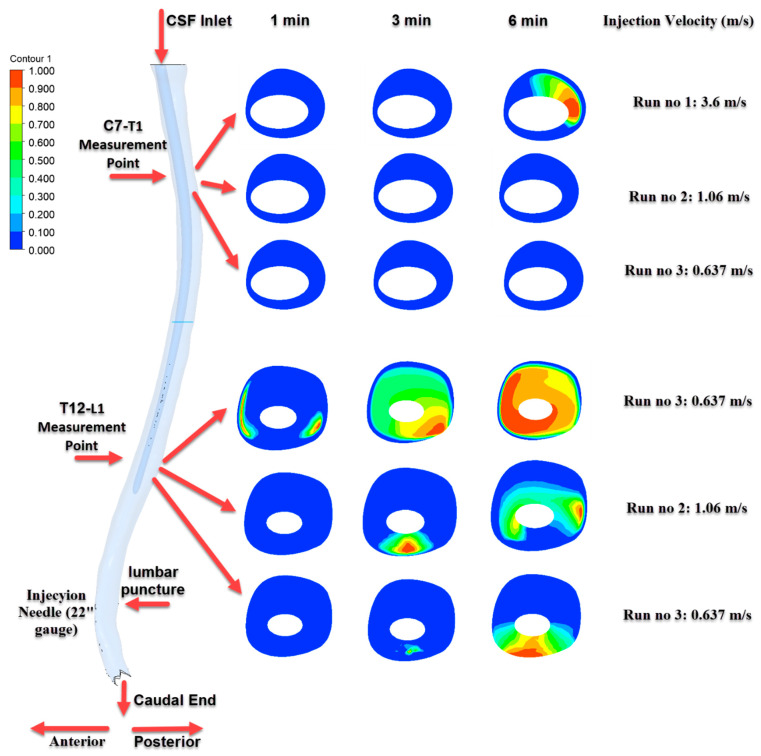
Time-dependent intrathecal drug distribution in the CSF for 22G needle injections.

**Figure 8:**
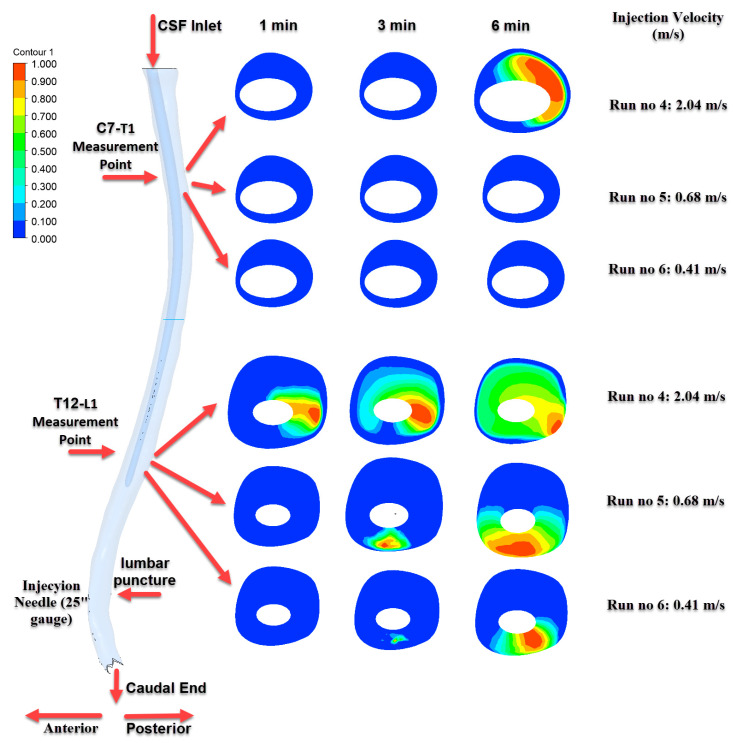
Time-dependent intrathecal drug distribution in the CSF for 25G needle injections.

**Figure 9:**
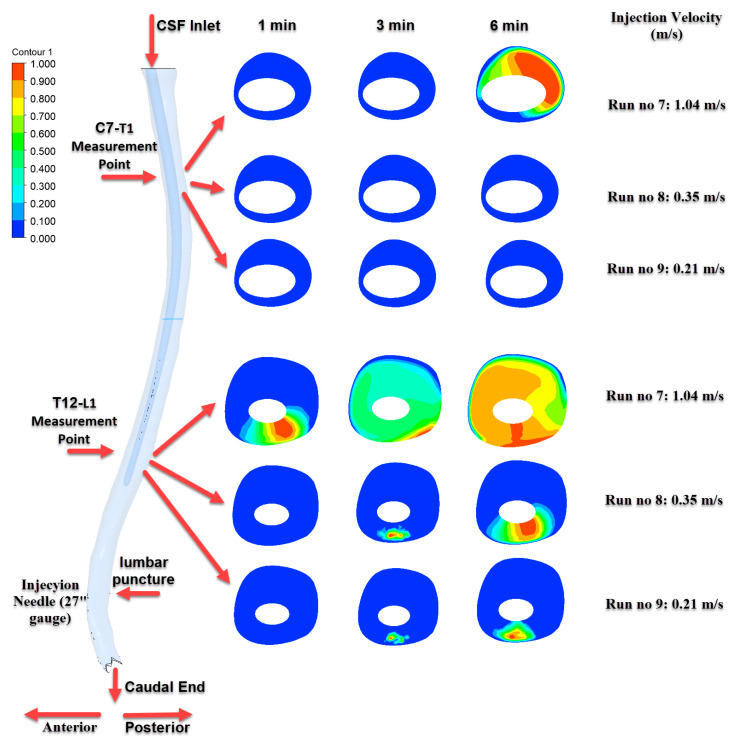
Time-dependent intrathecal drug distribution in the CSF for 27G needle injections.

Figure 7, Figure 8 and Figure 9 depict the time-dependent evolution of drug distribution in the cerebrospinal fluid (CSF) following intrathecal injections using 22G, 25G, and 27G needles, respectively. For each configuration, drug concentration contours were analyzed at 1, 3, and 6 min after the completion of injection. The results reveal a consistent pattern of progressive cranial transport over time. Notably, higher injection velocities facilitated earlier and broader spread toward the C7–T1 region, while lower velocities resulted in more localized retention around the T12–L1 level. These findings underscore the critical role of injection parameters in shaping both the spatial and temporal.

As shown in Figure 7, the most pronounced transport effect was observed in Run No: 1 (3.6 m/s) using a 22G needle. At the 6th minute, distinct concentration zones were detected at the C7–T1 level, demonstrating effective cranial transport of the drug. In contrast, for Run No: 2 and Run No: 3, the drug predominantly accumulated near the injection site and at the T12–L1 level, indicating more localized distribution.

Figure 8 illustrates the results of injections performed with a 25G needle. The highest distribution was observed in the Run No: 4 (2.04 m/s) configuration, where a progressively increasing and homogeneous distribution developed at the T12–L1 level over time. However, significant drug transport to the C7–T1 level was only detected at the 6th minute and only in this particular scenario.

Figure 9 corresponds to injections performed with a 27G needle. Due to the smaller needle diameter, lower injection velocities were applied. Under these conditions, although drug accumulation was observed in the T12–L1 region, concentrations reaching the C7–T1 level were not prominent except in Run No: 7. This finding indicates that cranial transport is limited when using finer needle gauges.

### 3.3. Drug Percentage Map

**Figure 10:**
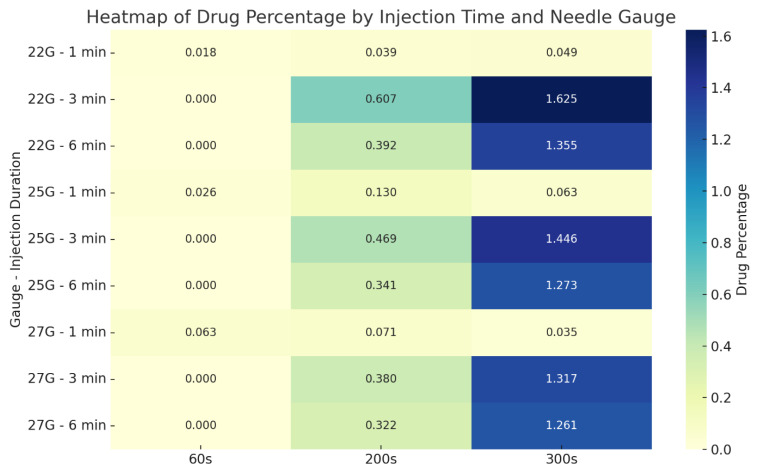
Heatmap showing average drug percentages within the CSF based on needle gauge and injection duration.

The heatmap presented in Figure 10 compares the average drug percentages within the CSF at 60, 200, and 300 s for all test scenarios. The results indicate that both longer injection durations and larger needle diameters lead to a significant increase in drug concentration over time.

The highest recorded drug percentage was 1.625% at 300 s in the experiment using a 22G needle with a 30-s injection duration. Similarly, the highest distribution in the 27G group was observed as 1.317%, also with a 30-s injection. These findings suggest that mid-duration (30 s) injections combined with larger gauge needles (22G, 25G) represent the most efficient configuration in terms of both transport and homogeneous distribution of the drug.

## 4. Discussion

In this study, the effects of injection parameters—namely needle gauge and injection velocity—on drug distribution within the CSF during spinal anesthesia were systematically investigated using a high-fidelity three-dimensional anatomical model and CFD methods. Our analysis focused on injections used at the L4–L5 vertebral level, evaluating the impact of different needle gauges (22G, 25G, and 27G) and injection velocities (0.01 m/s, 0.05 m/s, and 0.1 m/s) on the spatial distribution of drug concentration within the CSF.

By providing a detailed assessment of how these parameters influence drug dispersion, this study offers valuable insights for the optimization of spinal anesthesia practices, potentially improving both clinical effectiveness and patient safety.

The analysis results demonstrate that the use of a large-gauge needle (22G) combined with a high injection velocity (3.8 m/s) leads to sudden and elevated velocity values in the cerebrospinal fluid (CSF) near the injection site. This condition results in pronounced velocity gradients and elevated pressure levels within the local injection region [15,16]. This high-velocity flow enables the drug to initially propagate through the cerebrospinal fluid (CSF) with substantial kinetic energy, enhancing fluid mixing and convection. As a result, it facilitates the wider spatial distribution of the drug within the CSF [17,18]. In contrast, the use of smaller-gauge needles (27G) and lower injection velocities (e.g., 0.21 m/s) results in slower and more controlled velocity profiles. This leads to the drug remaining relatively localized near the injection site and prevents the formation of abrupt pressure gradients [15].

Moreover, high injection velocities combined with larger-gauge needles enhance convection and mixing mechanisms, promoting the transport of the drug to more distant cranial regions [14,17]. This may offer a clinical advantage in scenarios requiring rapid onset of action, such as emergency anesthesia or acute pain management [15]. However, the potential risk of high local drug concentrations leading to tissue toxicity or systemic side effects necessitates careful selection of these injection parameters [8]. Conversely, low injection velocities and smaller-gauge needles may offer a safer option in cases where a localized effect is desired; however, the limited cranial spread of the drug may pose a disadvantage in terms of therapeutic efficacy [16]. Additionally, it was observed that under high injection velocity and large-gauge needle conditions, the natural pulsations of the CSF (originating from cardiac and respiratory cycles) could be overridden by the drug’s kinetic effects, thereby facilitating its rapid cranial transport [1,19]. In contrast, under low injection velocity and small-gauge needle conditions, drug distribution remains largely dependent on the natural CSF flow, resulting in limited dispersion [8,17]. These findings indicate that high injection velocities enhance the convective transport within the CSF, thereby optimizing drug distribution. However, in injections performed with low velocities and small-gauge needles, the drug remains confined to a limited region near the injection site and fails to reach cranial segments effectively. Overall, the results clearly demonstrate that injection parameters have a direct impact on drug dispersion within the CSF and should be carefully selected in clinical practice based on specific therapeutic goals.

Injection duration also plays a critical role in drug distribution. Short-duration, high-velocity injections tend to produce a broader dispersion profile, whereas long-duration, low-velocity administrations increase the tendency of the drug to remain localized near the injection site [14]. Additionally, catheter orientation and injection location significantly influence flow dynamics within the CSF, thereby affecting the extent to which the drug reaches various spinal levels [17]. Properly directed catheters combined with high-velocity injections facilitate the transport of the drug to upper spinal segments, whereas misdirection or lower injection sites may limit its therapeutic effectiveness [1,17]. Flush applications have been shown to promote more homogeneous drug distribution within the CSF, particularly at low injection velocities; however, the effectiveness of this strategy appears to be highly dependent on anatomical configurations [20].

Our study has several limitations. First, the model was constructed based on MRI data from a single healthy individual; therefore, the impact of inter-individual anatomical variations on drug distribution could not be assessed. Second, all simulations were conducted in the supine position, and the effects of different patient postures (e.g., sitting position) on CSF flow and drug dispersion were not investigated. Additionally, the viscosity and density of the CSF were assumed to be constant based on literature values, without accounting for possible alterations in pathological conditions such as hydrocephalus. Furthermore, the simulation results have not yet been validated against experimental or clinical data. Lastly, pharmacological properties such as molecular weight, lipophilicity, diffusion coefficient, and protein binding were not included in the current CFD-based model. Future studies incorporating these parameters could enhance the predictive power and clinical applicability of simulation-based approaches.

## 5. Conclusions

This study demonstrated that injection parameters—particularly needle gauge (22G, 25G, 27G) and injection velocity (0.01 m/s, 0.05 m/s, 0.1 m/s)—have a significant impact on drug distribution within the cerebrospinal fluid (CSF) during spinal anesthesia. Analyses performed using an anatomically informed CFD model revealed that these parameters result in substantial differences in both local and cranial dispersion profiles.

Different combinations of injection parameters modulated the convective and diffusive mechanisms within the CSF, leading to distinct distribution patterns. Notably, the combination of a 22G needle with medium-to-high injection velocities (0.05–0.1 m/s) produced the most favorable distribution profile by ensuring widespread dispersion while minimizing the risk of excessive pressure buildup. In contrast, the use of a 27G needle with low injection velocity resulted in more localized and controlled distribution.

Consistent with existing literature, factors such as injection location, bolus volume, and post-injection flush were also shown to influence drug dispersion. Moreover, the inclusion of cardiac- and respiration-induced CSF pulsations in the model allowed for a more physiologically realistic simulation and highlighted their role in enhancing drug transport.

In this context, the findings underline the need for patient-specific customization of injection protocols in spinal anesthesia and intrathecal drug delivery. The CFD modeling approach provides a valuable framework to assist clinicians in developing individualized strategies that optimize therapeutic efficacy while minimizing potential risks.

## Figures and Tables

**Table 1:** Experiment parameters.

Run No	Needle Gauges	Injection Speed (m/s)	Injection Time (s)
1	22G	3.8	10
2	22G	1.06	30
3	22G	0.637	50
4	25G	2.04	10
5	25G	0.68	30
6	25G	0.41	50
7	27G	1.04	10
8	27G	0.35	30
9	27G	0.21	50

## Data Availability

No datasets were generated or analysed during the current study.

## Abbreviations

The following abbreviations are used in this manuscript:

CFD	Computational Fluid Dynamics
CSF	Cerebrospinal Fluid
HU	Hounsfield Unit
LP	Lumbar Puncture
MRI	Magnetic Resonance Imaging
PCMRI	Phase-Contrast Magnetic Resonance Imaging
PISO	Pressure-Implicit with Splitting of Operators
PRESTO!	Pressure Staggering Option
UPWIND	Upwind Differencing Scheme
